# The Book World of Medicine and Science

**Published:** 1898-10-29

**Authors:** 


### THE BOOK WORLD OF MEDICINE AND SCIENCE.

An Atlas of Bacteriology. Containing One Hundred and Eleven Original Photo-micrographs, with Explanatory Text. By Chas. Slater, M.A., M.B., M.R.C.S. Eng., F.C.S.; and Edmund J. Spitta, L.R.C.P. Lond., M.R.C.S. Eng., F.R.A.S. (London: Scientific Press. 1898. Price 7s. 6d. net.)

This handsome volume should prove a welcome addition to the literature of bacteriology, putting as it does into the hands of the student a reliable and most artistic presentment of the main phenomena which he will meet with in the course of his work in the bacteriological laboratory. The photographs which form the basis of this atlas are in every case original, and are taken from specimens such as are usually met with by the student in an ordinary course of bacteriology. As photo-micrographs they are worthy of the highest praise, and, of course, the specimens from which they are taken are typical, *i.e.*, have been carefully selected so as to represent the points which they are intended to illustrate. The aim, however, has evidently been not to make a collection of curious specimens, but to give a proper and fair representation of what may be expected to be produced by a first-class student who does his work with proper care. In this lies their extreme value as a guide and companion to those who are engaged upon this interesting subject. The book begins with two introductory chapters, one on certain photographic details of great interest to those engaged in like pursuits, and the other giving a general sketch of the organisms in question and of their classification. The main mass of the book is, however, made up of a series of plates, opposite each of which are given certain necessary details such as the name of the organism, the linear magnification, the lens by which it was viewed, and the stain employed in the preparation of the specimen, and along with these plates a series of chapters giving an account of each organism, its mode of occurrence, the disease which it produces, the forms in which it grows, and how it is best isolated and cultivated, especial attention being given to those other than mere morphological details by which these organisms can be identified and distinguished the one from the other. The book does not, of course, pretend to be a handbook of bacteriology. At the same time anything which can be seen is generally illustrated, and in every case photographic representations of the macroscopic appearances of the growths, whether in tubes or on plates, are given alongside of the photo-micrographs of the organisms themselves. In regard to the latter we have nothing but praise to give, the only criticism in which we can indulge being that the illustrations of growths within tissues are hardly equal to those of cultivations. This, however, is inevitable in monochrome, and in return one at any rate gets the picture untouched and unmodified, which is more than can be said for double colour printing. As a laboratory handbook and as a work of reference for medical officers of health and others, this atlas is likely to prove very useful.

Elements of Sanitary Law. By Alice Ravenhill, Lecturer to the National Health Society. With Introduction by Sir Richard Thorne, K.C.B., F.R.S., LL.D. (London: The Sanitary Publishing Company, Limited, 5, Fetter Lane, E.C. Price 6d.)

This little pamphlet of Miss Ravenhill's is one of the most useful books we have come across for a long time. Everyone needs at some time to know some part of sanitary law, and many people occasionally require to be acquainted with more than one. In this cheap little book they will find the substance of laws, which are sometimes elaborate and complicated in wording, stated with admirable clearness, and summarized most judiciously. Miss Ravenhill has undertaken a difficult but very important task, and has carried it out with the greatest success. We cordially recommend this sixpenny book to all who want to know by what sanitary laws they are governed.

An Atlas of Histology for the Use of Students; Being a Separate Issue of the 174 Original Coloured Illustrations from "A Text-Book of Histology." By Arthur Clarkson, M.B., C.M.Edin. (Bristol: John Wright and Co. 1898. Price 9s. net.)

In reviewing the text-book of histology from which these plates are taken, we have already expressed our high appreciation of their accuracy and artistic excellence, and we cannot but congratulate the students of the day on being able to obtain these beautiful illustrations of microscopic anatomy in so handy a form. Teaching may differ at the various schools, but the facts are the same, and as these plates are merely statements, although in a most luminous form, of the facts on which teaching is founded, they seem likely to be of service to the students of every school. We need not say more than that the book before us consists of 88 plates of chromo-lithographs, containing 176 figures of microscopic preparations, some of them being copies of selected bits of microscopic sections, while others are arranged more or less diagrammatically, so as to illustrate what under the microscope itself is not always very clear. It is likely to prove a very useful companion to those engaged in histological work.

The Health Resorts of Europe: A Medical Guide to the Mineral Springs, Climatic, Mountain, and Seaside Health Resorts, Milk, Whey, Grape, Earth, Mud, Sand, and Air Cures of Europe. By Thomas Linn, M.D. (London: Henry Kimpton. 1898. Price 2s. 6d. net.)

This little book on health resorts is a good one, and puts in simple language much that it is well for those who go about seeking for health to be aware of. Topical to the present season, when so many are going southwards for the winter, is the following: "As a rule we prefer night travelling for invalids. The trains are then of the best, many of them run sleepers, and it is best for a person in ill-health to get over a journey than to ride all day, perhaps on the sunny side of a carriage, and arrive tired at night to stop over in a strange hotel. We have found from long experience that one is more tired out by stopping over one night on the road than by going through. This, of course, refers to stopping one or two nights on the road, and not to the plan of taking a short day journey, and staying from three days to a week at each place. This delightful way of travelling is highly to be recommended." The book contains a therapeutic index giving the places which are imagined to be suitable for the various diseases and a description of the various health resorts mentioned arranged according to their countries. It seems to us to be a convenient little publication, and this is stated to be the sixth year of its issue.

